# Birth weight changes in a major city under rapid socioeconomic transition in China

**DOI:** 10.1038/s41598-017-01068-w

**Published:** 2017-04-21

**Authors:** Jian-Rong He, Wei-Dong Li, Min-Shan Lu, Yong Guo, Fan-Fan Chan, Jin-Hua Lu, Li-Fang Zhang, Song-Ying Shen, Xiao-Yan Xia, Ping Wang, Wei-Jian Mo, Kin Bong Hubert Lam, Jane E. Hirst, Hui-Min Xia, Xiu Qiu

**Affiliations:** 1 Division of Birth Cohort Study, Guangzhou Women and Children’s Medical Center, Guangzhou Medical University, Guangzhou, China; 2 Department of Woman and Child Health Care, Guangzhou Women and Children’s Medical Center, Guangzhou Medical University, Guangzhou, China; 3 Nuffield Department of Population Health, University of Oxford, Oxford, UK; 4 Nuffield Department of Obstetrics & Gynaecology, University of Oxford, Oxford, UK

## Abstract

Estimates of trends in birth weight may be useful in evaluating population health. We aimed to determine whether temporal changes in birth weight have occurred amongst 2.3 million neonates born in Guangzhou, China, during 2001–2015 and investigate the socioeconomic determinants of any changes. Trends in mean birth weight and annualized changes with the associated 95% confidence intervals (CIs) in the incidence of small for gestational age (SGA) and large for gestational age (LGA), defined as birth weight <10^th^ or >90^th^ centile respectively for gestational age and sex, were examined using linear and Poisson regression models. We found that mean birth weight declined by 1.07 grams/year from 2001 to 2015. After adjustment for gestational length, the decline in birth weight was attenuated (0.37 grams/year). The incidence of both SGA and LGA significantly decreased during the study period (annual decrease of 1.6% [95% CI, 1.5% to 1.7%] for SGA, 1.6% [95% CI, 1.5% to 1.8%] for LGA). We found a narrowing of disparities in SGA and LGA incidence across different maternal educational levels and residence location. Our results demonstrate that there has been an increase in the proportion of neonates born in the healthy birth weight range in Guangzhou.

## Introduction

China has made remarkable progress in improving maternal and child health and reducing maternal mortality, from 64 in 1996 to 22 per 100,000 in 2014 and infant mortality, from 36 in 1996 to 9 per 1000 live births in 2014^1^. Located in southern China, Guangzhou is one of the country’s most developed cities. Over the past two decades, a comprehensive healthcare system has been implemented, providing near universal health coverage to all citizens. The success of this system is reflected in the decline in maternal mortality rate, which at 7 per 100,000 in 2014, is far lower than the national average^2^.

Secular trends in birth weight can indicate changes in maternal health and nutrition. Babies with birth weights outside the normal range have higher risks of mortality and morbidity in the perinatal period and later in life^3, 4^. Small (SGA) or large (LGA) for gestational age are common indicators of restricted or excessive fetal growth. Previous studies have shown that SGA babies had increased risk of respiratory complications, hypoglycemia and necrotizing enterocolitis during perinatal period, neurological impairment in childhood and cardiovascular disease and type 2 diabetes mellitus in adulthood^5^, while LGA is associated with increased risk of birth trauma, cesarean section and postpartum hemorrhage as well as obesity, metabolic syndrome and type 2 diabetes mellitus in adulthood^3, 6^. Therefore, examining trends in incidence of SGA and LGA may be useful in evaluating population health. Conventionally, SGA or LGA have been defined as weight at birth below the 10^th^ or above the 90^th^ centiles when compared to a birth weight reference^7^. These references were based on the distribution of the weights of all babies born in a specific population, rather than describing healthy or physiological growth^7^. Recently, a multinational standard for newborn weight was published by the International Fetal and Newborn Growth Consortium for the 21^st^ Century (INTERGROWTH-21^st^)^7^. This study demonstrated that in mothers free from social, nutritional, medical and other constraints on fetal growth, the growth of babies around the world is remarkably similar. The INTERGROWTH-21^st^ birth weight standard therefore provides a valid instrument to compare fetal growth and birth weight throughout the world.

Data available on long-term trends of birth weight in China have been limited. We previously demonstrated in Guangzhou that babies born in 2012, when compared to 2002^8^, had lower mean birth weight with reduced incidence of SGA and LGA. It is possible however that this difference may not represent the actual long-term trends. We aimed to examine trends in mean birth weight and incidence of SGA and LGA among 2.3 million singleton births during 2001–2015 and whether these trends reflected changes in maternal characteristics including indicators of changing socio-economic status.

## Results

### Trends in maternal and infant characteristics

Maternal and infant characteristics by 3 discrete 5-year periods (2001–2005, 2006–2010, 2011–2015) are shown in Table 1. The proportion of older mothers (aged 35 years or older) almost doubled from 5.4% in 2001–2005 to 10.2% in 2011–2015. During this period, maternal educational attainment also improved, with 75% of mothers having completed at least senior high school qualifications. A quarter of the women in 2001–2005 were multiparous, rising to 43.7% in 2011–2015. The rate of cesarean delivery decreased from 41.6% in 2001–2005 to 37.1% in 2011–2015. The distribution of residence location and newborn sex ratio remained stable over this period.

**Table 1:** Maternal and newborn characteristics among singleton live births in Guangzhou, China from 2001 to 2015.

Characteristics	Birth year		
	2001–2005 (n = 439626)	2006–2010 (n = 759311)	2011–2015 (n = 1091808)
Mothers			
Age (years)^*^			
<25	117491 (27.0)	213066 (28.3)	256504 (23.6)
25–29	193968 (44.6)	314027 (41.7)	445965 (41.0)
30–34	99974 (23.0)	164549 (21.8)	273546 (25.2)
≥35	23690 (5.4)	61575 (8.2)	110746 (10.2)
Education^*,#^			
Low	205644 (46.8)	298349 (39.3)	188997 (25.8)
Medium	162434 (36.9)	324835 (42.8)	455226 (62.0)
High	71548 (16.3)	136127 (17.9)	89789 (12.2)
Parity^*^			
1	325976 (74.2)	511306 (67.3)	614617 (56.3)
2 or more	113578 (25.8)	247879 (32.7)	476580 (43.7)
Residence location			
Central area	175307 (39.9)	306113 (40.3)	445547 (40.8)
Suburban area	146526 (33.4)	260629 (34.3)	375713 (34.4)
Exurban area	117471 (26.7)	192569 (25.4)	270031 (24.8)
Newborns			
Sex			
Male	238433 (54.2)	410456 (54.1)	589515 (54.0)
Female	201193 (45.8)	348855 (45.9)	502293 (46.0)
Delivery modes^*^			
Vaginal delivery	255435 (58.1)	443709 (58.4)	685819 (62.8)
Assisted breech delivery	1458 (0.3)	1272 (0.2)	1143 (0.1)
Cesarean delivery	182730 (41.6)	314327 (41.4)	404844 (37.1)

Data are expressed as n (%).

^*^Missing data: maternal age (n = 15,907), education (n = 357,796), parity (n = 809) and delivery modes (n = 8).

^#^Education: low (junior school or less), medium (high school), high (College or above).

### Trends in birth weight and gestational length

Table 2 depicts yearly means of birth weight and gestational length, and the incidence of SGA and LGA during 2001–2015 in Guangzhou. Whilst mean newborn birth weight increased from 3191 grams in 2001 to 3212 grams in 2004, there was a decrease in 2009 to 3197 grams, with fluctuations between 2010 and 2015. Gestational length decreased from 39.02 weeks in 2001 to 38.84 weeks in 2015 (Table 2). During this 15-year period, the overall incidence of SGA, appropriate for gestational age (AGA) and LGA were 6.2%, 84.5%, and 9.3%, respectively; and when restricted to infants born preterm (gestational age at birth less than 37 weeks), the SGA and LGA incidence were 5.7% and 9.5%, respectively. The observed incidence of SGA in Guangzhou declined from 8.0% in 2001 to 5.6% in 2015, while that of LGA seemed to be stable at 8–9% (Table 2). Correspondingly, the observed incidence of AGA increased steadily from 83.1% in 2001 to 85.6% in 2015.

**Table 2:** Means of birth weight and gestational length and incidence of SGA and LGA during 2001–2015 in Guangzhou.

Birth year	Birth weight (grams) Mean (SD)	Gestational length (weeks) Mean (SD)	SGA, n (%)	LGA, n (%)
2001	3191 (432.5)	39.02 (1.45)	5297 (8.0)	5869 (8.9)
2002	3200 (433.0)	39.00 (1.44)	6204 (7.7)	7383 (9.2)
2003	3206 (434.1)	38.99 (1.44)	6400 (7.6)	8125 (9.6)
2004	3212 (433.9)	38.91 (1.43)	6677 (6.6)	10321 (10.2)
2005	3208 (433.7)	38.96 (1.43)	7526 (7.0)	10228 (9.6)
2006	3201 (433.4)	38.94 (1.43)	8454 (7.0)	11482 (9.5)
2007	3204 (430.9)	38.93 (1.41)	9834 (6.8)	13845 (9.6)
2008	3197 (428.6)	38.82 (1.40)	10116 (6.4)	15319 (9.7)
2009	3192 (427.0)	38.86 (1.40)	10182 (6.5)	14576 (9.3)
2010	3196 (422.2)	38.83 (1.38)	10702 (6.0)	16298 (9.2)
2011	3190 (422.7)	38.83 (1.37)	12213 (6.1)	17828 (9.0)
2012	3196 (421.4)	38.73 (1.35)	12464 (5.4)	22550 (9.7)
2013	3191 (421.6)	38.75 (1.35)	11661 (5.5)	19347 (9.1)
2014	3194 (419.4)	38.79 (1.34)	12647 (5.5)	20631 (9.0)
2015	3191 (419.4)	38.79 (1.34)	12222 (5.6)	19410 (8.8)
Overall	3196 (425.7)	38.84 (1.39)	142599 (6.2)	213212 (9.3)

SGA, small for gestational age; LGA, large for gestational age, based on the INTERGROWTH-21st standard.

Table 3 shows the change per year in birth weight and gestational length and the incidence of SGA and LGA. In the crude model using birth year as a linear predictor, birth weight declined by 1.07 grams/year, with a steeper slope after adjustment for maternal age at delivery, parity and infant sex (2.70 grams/year), and was largely attenuated but not eliminated (0.37 grams/year) after further adjusting for gestational length (Table 3). This decrease in birth weight appeared to be more pronounced among boys (0.48 grams/year; 95% CI, 0.31 to 0.65 grams/year) than girls (0.24 grams/year; 95% CI, 0.06 to 0.42 grams/year). There was also a trend for shorter gestational length, after adjustment for maternal age, parity and infant sex (−0.02 week/year). Considering birth weight for gestational age, the incidence of both SGA and LGA significantly decreased. After adjustment for maternal age, parity, infant sex and gestational length, the decreasing trend in SGA incidence was less pronounced but remained significant, while that in LGA incidence was strengthened (annual decrement of 1.6% [95% CI, 1.5% to 1.7%] for SGA, 1.6% [95% CI, 1.5% to 1.8%] for LGA) (Table 3). The results did not change substantially when the analysis was restricted to term birth babies.

**Table 3:** Change per year in incidence of SGA and LGA and birth weight and gestational length.

Outcome	Model 1	Model 2	Model 3
β (95% CI)			
Birth weight (grams)	−1.07 (−1.21, −0.94)	−2.70 (−2.84, −2.56)	−0.37 (−0.49, −0.25)
Gestational length (weeks)	−0.02 (−0.020, −0.02)	−0.02 (−0.02, −0.02)	—
RR (95% CI)			
SGA	0.973 (0.972, 0.974)	0.980 (0.979, 0.981)	0.984 (0.983, 0.985)
LGA	0.995 (0.994, 0.997)	0.985 (0.984, 0.986)	0.984 (0.982, 0.985)

SGA, small for gestational age; LGA, large for gestational age, based on the INTERGROWTH-21st standard.

Model 1, no adjustment.

Model 2, adjusted for mother’s age at delivery, parity and infant sex.

Model 3, adjusted for variables as in model 2 plus gestational length.

### Stratified trends in incidence of SGA and LGA

**Figure 1:**
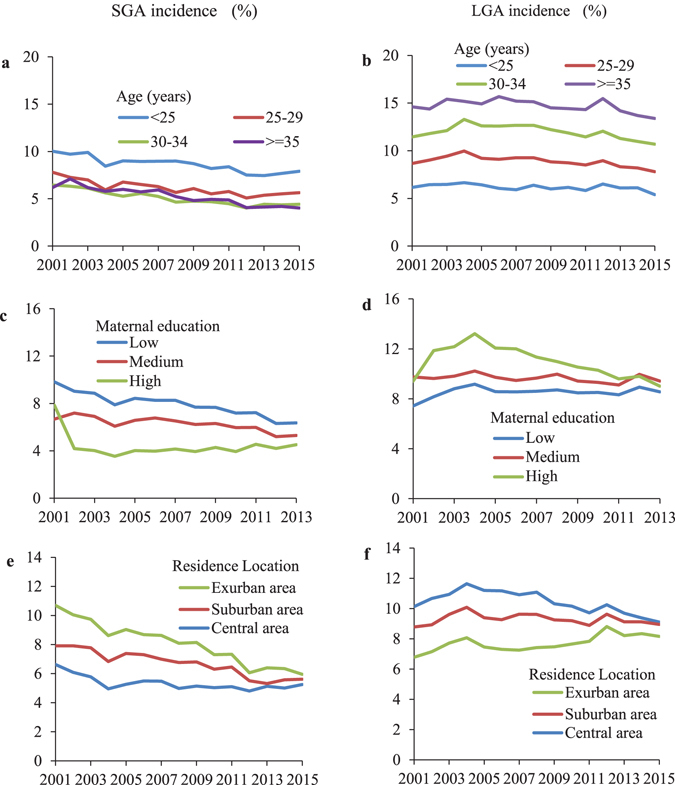
Changes in the incidence of SGA (left panel) and LGA (right panel) based on maternal age group (**a**,**b**), education level (**c**,**d**), and residence location (**e**,**f**) during 2001–2015. SGA, small for gestational age; LGA, large for gestational age.

Figure 1 presents the stratified trends in incidence of SGA and LGA. Newborns of younger mothers (<25 years), with lower educational attainment and living in ex-urban areas had increased risk of SGA while a decreased risk of LGA (Fig. 1). The trends in incidence of SGA and LGA among the other maternal age groups were very similar. Newborns of mothers with lower education level tended to have more evident decreasing trend in SGA incidence (Fig. 1c), whereas those of mother with high education had more evident decreasing trend in LGA incidence (Fig. 1d). The decreasing trend in SGA incidence was more pronounced among mothers who lived in exurban area (Fig. 1e), while the downward trend in LGA incidence appeared to be more evident among mothers who lived in the central area (Fig. 1f). After adjusting for maternal and infant characteristics, all subgroups had decreasing trends in SGA and LGA incidence (Supplementary Table 1). Table 4 shows the risk of SGA and LGA by maternal characteristics in selected years (2001, 2005, 2010 and 2015). The risk ratios (RRs) for each subgroup of maternal education and residence location tend to become null in later years (Table 4). Significant interactions between birth year and maternal education and residence locations were observed (P < 0.001), suggesting a narrowing of the disparities in SGA and LGA risks across different maternal educational levels and residence locations.

**Table 4:** Risk of SGA and LGA by maternal characteristics in selected years.

Birth weight for gestational age	2001		2005		2010		2015	
	%	RR (95% CI)^#^	%	RR (95% CI)^#^	%	RR (95% CI)^#^	%	RR (95% CI)^#^
SGA								
Maternal age (years)								
<25	10.0	Reference	9.0	Reference	8.2	Reference	7.9	Reference
25–29	7.8	0.803 (0.754, 0.855)	6.8	0.796 (0.755, 0.839)	5.5	0.717 (0.686, 0.749)	5.7	0.761 (0.728, 0.795)
30–34	6.5	0.708 (0.651, 0.770)	5.3	0.663 (0.618, 0.710)	4.7	0.644 (0.608, 0.683)	4.4	0.667 (0.633, 0.703)
≥35	6.2	0.754 (0.644, 0.883)	6.0	0.809 (0.725, 0.901)	4.9	0.733 (0.676, 0.795)	4.0	0.677 (0.630, 0.727)
Education								
Low	9.8	Reference	8.4	Reference	7.2	Reference	6.4	Reference
Medium	6.7	0.658 (0.617, 0.701)	6.6	0.752 (0.716, 0.790)	6.0	0.812 (0.779, 0.846)	5.3	0.775 (0.741, 0.811)^*^
High	7.9	0.792 (0.740, 0.848)	4.0	0.447 (0.411, 0.485)	3.9	0.493 (0.461, 0.528)	4.5	0.606 (0.562, 0.654)^*^
Residence Location								
Exurban area	10.7	Reference	9.0	Reference	7.3	Reference	6.0	Reference
Suburban area	7.9	0.739 (0.691, 0.791)	7.4	0.826 (0.783, 0.872)	6.3	0.855 (0.816, 0.896)	5.6	0.922 (0.881, 0.966)
Central area	6.6	0.602 (0.564, 0.643)	5.3	0.583 (0.551, 0.617)	5.0	0.676 (0.644, 0.709)	5.3	0.832 (0.795, 0.870)
LGA								
Age (years)								
<25	6.2	Reference	6.4	Reference	6.2	Reference	5.4	Reference
25–29	8.7	1.369 (1.273, 1.472)	9.2	1.366 (1.294, 1.443)	8.7	1.351 (1.293, 1.411)	7.8	1.371 (1.308, 1.437)
30–34	11.5	1.697 (1.564, 1.841)	12.6	1.738 (1.638, 1.845)	11.9	1.742 (1.661, 1.827)	10.7	1.721 (1.639, 1.807)
≥35	14.6	2.016 (1.793, 2.267)	14.9	1.952 (1.803, 2.114)	14.4	1.992 (1.881, 2.110)	13.4	2.025 (1.918, 2.139)
Education								
Low	7.4	Reference	8.6	Reference	8.5	Reference	8.6	Reference
Medium	9.8	1.426 (1.338, 1.519)	9.7	1.226 (1.174, 1.281)	9.3	1.158 (1.118, 1.200)	9.4	1.192 (1.148, 1.238)^*^
High	9.4	1.310 (1.223, 1.404)	12.1	1.699 (1.607, 1.797)	10.3	1.487 (1.417, 1.562)	9.0	1.278 (1.208, 1.353)^*^
Residence Location								
Exurban area	6.8	Reference	7.5	Reference	7.7	Reference	8.2	Reference
Suburban area	8.8	1.295 (1.202, 1.396)	9.4	1.226 (1.162, 1.293)	9.2	1.190 (1.140, 1.242)	9.0	1.117 (1.075, 1.160)
Central area	10.1	1.611 (1.502, 1.728)	11.2	1.555 (1.477, 1.636)	10.2	1.391 (1.335, 1.450)	9.1	1.183 (1.140, 1.227)

^*^Results for educational level were obtained using data in 2013. ^#^Adjusted for variables as in model 2 plus gestational length.

## Discussion

In one of the most developed cities in China, we found mean birth weight declined by 1.07 grams/year from 2001 to 2015. This decline was largely explained by shorter gestational length. We have demonstrated that during this same time, both SGA and LGA incidence decreased (annual decrease of 1.6% [95% CI, 1.5% to 1.7%] for SGA, 1.6% [95% CI, 1.5% to 1.8%] for LGA), which cannot be entirely explained by changes in maternal and infant characteristics. By using education and residence location as proxies for maternal socio-economic status, we present evidence that the disparities in SGA and LGA risk between mothers with different socio-economic status has narrowed.

Studies of secular trends of birth weight in developed countries have demonstrated birth weight has begun to decline during the last three decades^9–15^. In the United States, mean birth weight among term singleton neonates decreased from 3441 grams in 1990 to 3389 grams in 2004 (3.0 grams/year), independent of decreasing gestational length^12^. Such data from China have been relatively sparse and mostly short-term. An analysis from southeast China found the mean birth weight for term and post-term infants decreased from 3378 grams in 2000 to 3369 grams in 2005^16^. By contrast, a recent study in Shaanxi province (northwestern China) reported the rates of low birth weight decreased while those of macrosomia increased, indicating an increase in higher birth weights in this population^17^. Our data demonstrate that there has been a decrease in mean birth weight in Guangzhou. Gestational age is a strong determinant of birth weight; thus changes in gestational age at birth may result in trends in birth weight. Over the 15-year study period, we observed mean gestational length in Guangzhou decreased from 39.02 to 38.79 weeks (1.6-day difference). After adjustment for gestational length, the downward trend of mean birth weight was largely attenuated, suggesting the shortening of gestation length was likely to be responsible for the observed decrease in mean birth weight, which is in contrast to the observations from the United States^12^.

We observed that the incidence of SGA and LGA in our population decreased over time. After adjusting for maternal age, parity, infant sex and gestational length, the decreasing trend of SGA incidence was attenuated but remained significant, while that of LGA incidence was strengthened, indicating these covariates did not, at least not fully, explain the decline in incidence of SGA and LGA. A plausible explanation of the observed trend is the improvement of maternal care, resulting from a variety of public health programs in Guangzhou that aimed to promote maternal and child health (Supplementary Fig. 1). In 2001 and 2011 two 10-year Development Guidelines for Women and Children’s Health were set by the local government, respectively, aiming to improve reproductive health services and birth outcomes, and setting specific targets for maternal health. These targets included: increasing the proportion of pregnant women receiving systematic management (establishment of health records, ≥5 times prenatal visit, delivery in hospital and ≥1 postpartum visit) to 95% or above by 2015; reducing rates of maternal moderate-to-severe anemia; and reducing rates of low birth weight. Other public health measures (Supplementary Fig. 1), including regular obstetric care quality assessment, clinical training for obstetricians and improved management of high-risk pregnancies, have also contributed to the quality of maternal care. Data from Guangzhou Maternal Health Surveillance Annual Report showed that rates of pregnant women receiving systematic management increased from 78.7% in 2001 to 95.9% in 2015, rates of starting the prenatal visit at first trimester increased from 81.9% in 2001 to 96.7% in 2015, and rates of maternal moderate-to-severe anemia decreased from 1.36% in 2008 to 0.29% in 2015 (data not published), supporting the effectiveness of public health measures and improvement of maternal care in Guangzhou.

In our previous study, we found the mean birth weight in 2012 was 25 grams lower than that in 2002 (roughly equal to a 2.27 grams/year)^8^. This magnitude of the decrease appeared to be stronger than that observed in current study (1.07 grams/year). On the other hand, the decreasing trend of SGA incidence in current analysis is more evident (from 8.0% in 2001 to 5.6% in 2015) than that in our previous report (9.2% in 2002 and 8.6% in 2012). These differences might be due to the fact that in the current analysis we have included a much larger dataset (2.3 million babies) than previous study (<90 thousand babies) and used the INTERGROWTH-21^st^ standard to define SGA and AGA instead of local birth weight reference^8^. In the current study we also found narrowing disparities in incidence of SGA and LGA across maternal educational levels and residence locations. This might also be attributed by the implementation of above-mentioned public health programs in Guangzhou (Supplementary Fig. 1), which improved the equality for access to antenatal care service. Our findings suggest that the gap in birth weight outcomes across social classes could be closed by removing the barriers of access to healthcare services for the less privileged population, which has important public health implications in other parts of China and elsewhere.

The major strength of present study is the use of a large (over 2.2 million births) and up-to-date (until 2015) dataset covering a sufficiently long period of time (15 years) to examine the trends in birth weight. In addition, we used the INTERGROWTH-21^st^ standard to define SGA and LGA. To the best of our knowledge, until now this new standard has not been used to inspect temporal trends in birth weight in any population. Some studies have advocated the continued use of local or customized charts^18, 19^; however these local charts are only relevant to the population and time from which they were derived and hence make comparison between populations and studies impossible.

Some limitations need to be mentioned. Firstly, because the Guangzhou Perinatal Health Care and Delivery Surveillance System (GPHCDSS), from which we obtained the data, was not designed for research purposes, some variables relevant to fetal growth were not collected. This meant that we were unable to examine the contribution of pre-pregnancy body mass index (BMI), gestational weight gain, maternal diet and smoking^20^. Secondly, although it is standard practice to confirm gestational age using ultrasound examination during first or early second trimester in Guangzhou^21^, the last menstrual period-based method was also used in some rural areas, which may have resulted in miscalculation of gestational age. Lastly, we have no data on length and head circumference at birth, two other important indicators of fetal growth, in this dataset.

In conclusion, although mean birth weight decreased slightly, we observed a substantial improvement in key fetal growth indicators (SGA and LGA) in Guangzhou during 2001–2015. This occurred in parallel to improvements in maternal health care. Birth size is a strong predictor of not only short-term health outcome but also long-term consequences during adulthood. Optimization of birth weight for gestational age could have a significant influence on child health. By sharing the experience learnt in Guangzhou, we hope that this improvement may be replicated as other regions in China and other low- and middle-income countries.

## Methods

### Data Source and Study Population

Birth data between 2001 and 2015 were acquired from the Guangzhou Perinatal Health Care and Delivery Surveillance System (GPHCDSS), which has been described elsewhere^8, 22, 23^. In brief, the electronic GPHCDSS database was implemented in 2000, and covers over 99% of deliveries in Guangzhou (population: 13 million). Dedicated trained health workers are responsible for collating and registering birth information at each hospital. Validity was confirmed at various levels: by the Chief Midwife, the Chief Physician, and the Department of Medical Administration at each hospital; by the newborns’ parents at the time when the birth certificate was issued; and by the Guangzhou Municipal Health Bureau through annual sampling survey. From this surveillance system information on maternal demographics, pregnancy and delivery was collected.

This analysis included all singleton live births delivered in Guangzhou from 2001 to 2015, with gestational ages between 33 and 42 completed weeks of gestation that in line with the INTERGROWTH-21^st^ Birth Weight Standards^7^. We also obtained information from the GPHCDSS dataset on maternal and newborn characteristics, including maternal age at delivery and educational level, parity, delivery mode, date of birth, newborn’s sex, gestational age and birth weight.

A total of 2,388,629 records of live births born in 2001–2015 were retrieved and 97,884 were excluded due to duplicate records (n = 8826), multiple pregnancy (n = 63,530), missing or implausible data on birth weight (n = 1274), sex (n = 31), and gestational age (n = 999), gestational age of less than 33 weeks (n = 18,414), or more than 42 weeks (n = 4,810). As a result 2,290,745 records of singleton live births were included in the analysis. This study was conducted in compliance with local and national regulations and was approved by the institutional ethical committee board of Guangzhou Women and Children’s Medical Center. Data used in this study were anonymous, and no individual identifiable information was available for the analysis.

### Measurements and Definitions of SGA and LGA

Birth weight (in grams) was measured routinely by registered midwives using an electronic weighing scale within half an hour of delivery. Gestational age at birth was determined based on an ultrasound examination in the first or second trimester and was expressed as completed weeks. Where ultrasound data were unavailable, the last menstrual period was used to calculate gestational age. We defined SGA or LGA as birth weight below the 10^th^ or above 90^th^ centile of a sex- and completed gestational age specific birth weight, according to the published INTERGROWTH-21^st^ standard^7^. Infants having birth weight between 10^th^ and 90^th^ centiles were considered to be AGA.

### Statistical Analyses

Descriptive statistics of maternal and infant characteristics are shown in 3 discrete 5-year periods (2001–2005, 2006–2010 and 2011–2015), including maternal age (<25, 25–29, 30–34 and ≥35 years), educational level (low: junior school or less; medium: high school; high: college or above), parity (1 and ≥2), residence location (central area, suburb area and exurban area), delivery mode (vaginal delivery, assisted breech delivery and cesarean delivery) and infant sex (male and female). We then calculated the mean values of birth weight, gestational length, the incidence of SGA and LGA for each year between 2001 and 2015. Trends in birth weight and gestational length assessed using linear regression models, with birth year included as a continuous variable, and trends in SGA and LGA were examined using Poisson regression models with robust variance to estimate risk ratios (RRs) and 95% confidence intervals (CIs) for the change per year. We presented 3 models to explore the trends in outcomes: Model 1 was unadjusted to quantify the crude trend; Model 2 was adjusted for maternal age, parity and infant sex; Model 3 was further adjusted for gestational length that had a decreased trend. If changes in covariates accounted for the observed trends in the outcomes, adjustment for these variables would attenuate the estimated RRs of birth year. We did not adjust for maternal education in the model because data on education level had not been routinely collected since 2014 and the estimates of RR changed only slightly when included education level in the model.

We then presented the trends in incidence of SGA and LGA graphically, stratifying by maternal age groups, educational level and residence location, respectively. To describe the difference in risk of SGA and LGA among these subgroups, we calculated RRs and 95% CIs of each subgroup for each year separately. We also included multiplicative interaction terms between birth year and maternal age groups, educational level, residence location in Model 3 separately to test whether the difference in risk of SGA and LGA among these subgroups differed over the 15-year period.

We used SAS version 9.2 (SAS Institute, Cary, NC) for all statistical analyses.

### Data availability

The data that support the findings of this study are available from Guangzhou Women and Children’s Health Information Center but restrictions apply to the availability of these data, which were used under license for the current study, and so are not publicly available. Data are available from the authors upon reasonable request and with permission of Guangzhou Women and Children’s Health Information Center (contact Xiao-Yan Xia at xiaxy0656@163.com).

## Electronic supplementary material

Supplementary Information
