# Supplementary material for: Birth weight changes in a major city under rapid socioeconomic transition in China
Source: Sci Rep. 2017 Apr 21;7:1031. doi: 10.1038/s41598-017-01068-w (PMC5430650; doi:10.1038/s41598-017-01068-w)
Supplement: Supplementary file 1 — Supplementary Information [file 41598_2017_1068_MOESM1_ESM.pdf]

Supplementary Information

Birth weight changes in a major city under rapid socioeconomic transition in China

Jianrong He[1, †], Weidong Li[1, †], Minshan Lu[1, †],Yong Guo[1], Fanfan Chan[1], Jinhua Lu[1], Lifang Zhang[1], Songying Shen[1], Xiaoyan Xia[1], Ping Wang[1], Weijian Mo[1], Kin Bong Hubert Lam[2], Jane E Hirst[3], Huimin Xia[1, *], Xiu Qiu[1, *]

[*] Contributed equally

[1]Division of Birth Cohort Study, Guangzhou Women and Children's Medical Center, Guangzhou Medical University, Guangzhou, China

[2]Nuffield Department of Population Health, University of Oxford, Oxford, UK

[3]Nuffield Department of Obstetrics & Gynaecology, University of Oxford, Oxford, UK

[*] Corresponding author: Dr Xiu Qiu, Division of Birth Cohort Study and Department of Woman and Child Health, Guangzhou Women and Children's Medical Center, Guangzhou Medical University, Guangzhou 510623, China, telephone: +86 20 38367162 fax: +86 20 38367162 qxiu0161@163.com; or Prof Huimin Xia, Division of Birth Cohort Study and Department of Neonatal Surgery, Guangzhou Women and Children's Medical Center, Guangzhou Medical University, Guangzhou 510623, China, telephone: +86 20 38076019 fax: +86 20 38076019 huimin.xia876001@gmail.com

Supplementary Figure

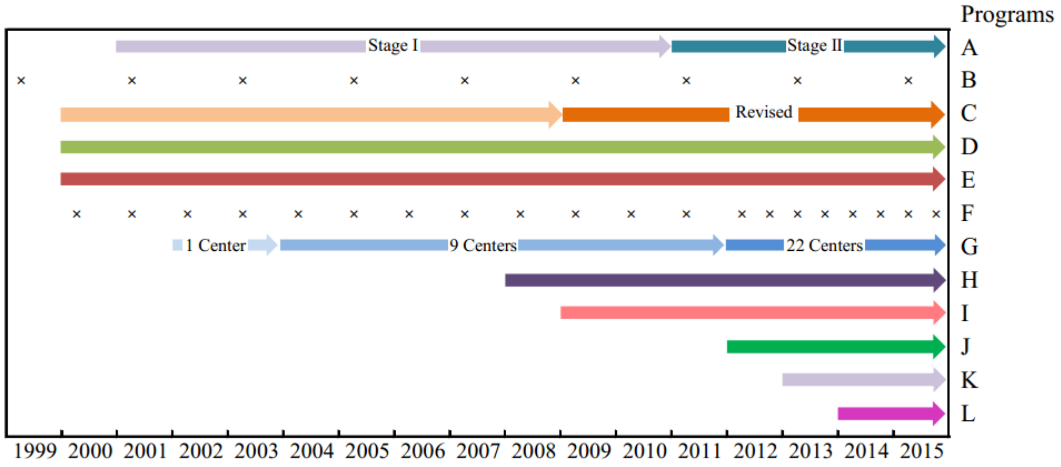

Supplementary Figure 1. Implemented programs for promotion of maternal health in Guangzhou. A, Development Guidelines of Women and Children in Guangzhou; the first stage covered 2001-2010 and the second stage covered 2011-2020. B, Obstetric care quality assessment at municipal level. C, Management of high-risk pregnancy. D, Training of newly recruited obstetricians. E, Standardized clinical training for existing obstetricians. F, Regular meetings of maternity unit directors in Guangzhou. G, Establishment of Obstetric Critical Care Centers. H, Promotion of systematic management of maternal and child health care. I, Promotion of systematic management of perinatal health. J, Maternal and Child Health Action Plan. K, Clinical training for 200 obstetricians and 100 midwives in rural areas. L, Establishment and implementation of the education platform for maternal and child health.

Supplementary Table:

Supplementary Table 1. Temporal trends of SGA and LGA among different subgroups

Characteristics	SGA		LGA	
	Crude RR (95% CI)	Adjusted RR (95% CI) *	Crude RR (95% CI)	Adjusted RR (95% CI) *
Maternal age				
<25	0.981 (0.979, 0.983)	0.989 (0.987, 0.992)	0.993 (0.991, 0.996)	0.985 (0.983, 0.988)
25-29	0.978 (0.976, 0.980)	0.985 (0.984, 0.987)	0.989 (0.987, 0.990)	0.982 (0.981, 0.984)
30-34	0.971 (0.968, 0.974)	0.978 (0.975, 0.980)	0.989 (0.987, 0.991)	0.985 (0.983, 0.987)
≥35	0.961 (0.956, 0.966)	0.967 (0.962, 0.973)	0.991 (0.988, 0.994)	0.986 (0.983, 0.989)
Education				
Low	0.965 (0.963, 0.967)	0.983 (0.981, 0.985)	1.006 (1.004, 1.009)	0.988 (0.986, 0.990)
Medium	0.973 (0.971, 0.975)	0.990 (0.988, 0.992)	1.000 (0.998, 1.002)	0.981 (0.979, 0.982)
High	0.978 (0.973, 0.982)	0.989 (0.984, 0.994)	0.987 (0.984, 0.990)	0.974 (0.971, 0.977)
Residence Location				
Central area	0.989 (0.987, 0.991)	0.997 (0.995, 0.999)	0.986 (0.984, 0.987)	0.974 (0.973, 0.976)
Suburban area	0.971 (0.969, 0.974)	0.983 (0.981, 0.985)	0.997 (0.995, 0.999)	0.986 (0.984, 0.988)
Exurban area	0.960 (0.958, 0.962)	0.975 (0.973, 0.977)	1.012 (1.010, 1.014)	0.997 (0.994, 0.999)

* Adjusted for gestational length, mother's age at delivery, parity and newborn sex, except for the stratifying variable.
